# Stress-Related Immunomodulation of Canine Lymphocyte Responses and Hematologic Profiles

**DOI:** 10.3390/ijms27031506

**Published:** 2026-02-03

**Authors:** Marek Kulka, Iwona Monika Szopa, Karolina Mizera-Szpilka, Maciej Klockiewicz

**Affiliations:** 1 Department of Pathology and Veterinary Diagnostics, Institute of Veterinary Medicine, Warsaw University of Life Sciences, 02-776 Warsaw, Poland; 2 Department of Physiological Sciences, Institute of Veterinary Medicine, Warsaw University of Life Sciences, 02-776 Warsaw, Poland; iwona_szopa@sggw.edu.pl; 3 Department of Infectious Diseases and Public Health, Faculty of Veterinary Medicine, University of Agriculture in Kraków, 30-248 Krakow, Poland; karolina.mizera@urk.edu.pl; 4 Department of Preclinical Sciences, Institute of Veterinary Medicine, Warsaw University of Life Sciences, 02-786 Warsaw, Poland; maciej_klockiewicz@sggw.edu.pl

**Keywords:** memory phenotype, chronic stress, apoptosis, hematology

## Abstract

The immune status of dogs is shaped by continuous exposure to antigenic and various environmental stimuli, which together influence the development, regulation, and effectiveness of immune responses. Stress-related immune alterations may not be evident at the systemic level but can emerge at cellular and molecular scales. Therefore, this study aimed to comprehensively characterize the hematological and immunological profiles of dogs in different environments. We evaluated lymphocyte responses under basal conditions and following CD3/CD28-mediated in vitro activation, with subsequent long-term culture. Gene expression analyses targeted markers of early T cell activation, cytotoxic effector function, cytokine signaling, and inhibitory immune regulation. The memory phenotype of T lymphocytes was evaluated after blood collection and prolonged in vitro culture. In addition, hematological and biochemical profiles were assessed, including basic parameters, cortisol, and C-reactive protein. Our results revealed that client-owned dogs exhibited lower baseline expression of activation markers, especially in comparison with the short-term stay group, indicating an early immune activation state upon entry to the shelter environment. Furthermore, T lymphocytes from short- and long-term shelter dogs exhibited marked differences in the distribution of naïve and effector-memory subsets as well as different expansion capacity. These alterations persisted during prolonged in vitro culture, indicating that stress duration and environmental antigen exposure differentially shape immune responsiveness. In summary, chronic stress modulates canine immune status in a time-dependent manner, highlighting the importance of integrated cellular and molecular approaches in assessing the impact of environmental stressors on dogs’ health and welfare.

## 1. Introduction

Many studies have demonstrated the impact of stress on the hematological profile. One of the most common stress-related changes is the development of a stress leukogram, observed in conditions associated with elevated cortisol levels [1,2,3], characterized by neutrophilia and monocytosis accompanied by lymphopenia. Lowered lymphocyte count in blood morphology may result from lymphocytes’ redistribution from blood to lymphoid tissues and, when stress-related conditions become more prolonged, also from increased lymphocyte apoptosis. Chronic exposure to stress has been shown to reduce the proliferation of both T and B lymphocytes, leading to impaired adaptive immune responses and increased susceptibility to infectious diseases [4,5]. Individuals subjected to long-term stress exhibit a marked decrease in T lymphocyte counts, which further compromises immune competence [6]. However, stress-induced immune alterations may be more subtle and detectable only at the cellular and molecular levels, as presented in our previous study [7].

T lymphocytes play a central role in the immune system by identifying and responding to specific antigens, with helper T cells (CD4^+^) coordinating the activity of other immune cells and cytotoxic T cells (CD8^+^) directly eliminating infected or abnormal cells [8,9]. Helper T cells participate in recognizing antigens presented by antigen-presenting cells, such as dendritic cells, macrophages, and B cells, in association with MHC class II molecules [10,11]. Upon activation, CD4^+^ T lymphocytes secrete various cytokines that modulate the activity of other immune cells. These include regulating B-cell antibody production, enhancing the cytotoxic activity of CD8^+^ T cells, and promoting the phagocytic function of macrophages. In addition, CD4^+^ T cells help maintain immune system balance by supporting regulatory T cell functions and preventing excessive immune responses that could lead to autoimmune diseases [11,12]. Cytotoxic T cells recognize specific antigens presented on the surface of infected, transformed, or cancerous cells in the context of MHC class I molecules. Following recognition, they release cytotoxic effector molecules such as perforin, which forms pores in the target cell membrane, and granzymes, which enter target cells and trigger apoptosis [13,14]. This precise mechanism is essential for the control of viral infections—such as canine distemper virus and parvovirus—as well as for the immune surveillance and elimination of tumor cells [15,16]. In addition to their cytotoxic functions, CD8^+^ T cells can produce proinflammatory cytokines, such as interferon-gamma (IFN-γ), further enhancing immune responses [17,18].

The T lymphocyte population comprises several functionally distinct subsets, including naïve T cells, central-memory T cells (T_CM_), and effector-memory T cells (T_EM_). Naïve T cells are mature T lymphocytes that have not yet encountered their specific antigen. They circulate through the blood and secondary lymphoid organs, such as lymph nodes and the spleen, where they are primed to respond upon first antigen exposure. Following activation, some naïve T cells differentiate into long-lived memory populations. Centralmemory resides mainly in lymphoid tissues and is characterized by high proliferative capacity and rapid responsiveness upon antigen re-encounter. The classification of T lymphocyte subpopulations varies across species. In dogs, T cell subsets are commonly defined based on the differential expression of surface markers CD44 and CD62L. As demonstrated by Rothe et al. (2017) [19], the combined expression patterns of these two molecules allow for the identification of three distinct T lymphocyte subsets: naïve T cells (T_Naïve_, CD44^low^CD62L^high^), central-memory T cells (T_CM_, CD44^high^CD62L^high^), and effector-memory T cells (T_EM_, CD44^high^CD62L^low^). CD62L (L-selectin) is expressed on naïve and central-memory T cells and is rapidly downregulated following T cell activation, making it a key marker for distinguishing central-memory (CD62L^+^) from effector-memory (CD62L^−^) subsets. The regulation of CD62L is essential for controlling T lymphocytes’ trafficking between peripheral tissues and secondary lymphoid organs [20]. In contrast, CD44 is upregulated upon antigen-driven activation of naïve T cells and remains highly expressed on memory T lymphocytes, which are responsible for mediating secondary immune responses [21].

Dogs housed in shelters or kennel facilities are often exposed to chronic stress as a result of environmental conditions that may not fully accommodate their natural behavioral patterns and physiological needs [22,23,24]. While basic hematological and behavioral assessments provide some insight into welfare, more sensitive approaches are required to effectively evaluate immune function. Therefore, this study aimed to characterize lymphocyte functions in dogs living in different environments. Our investigation focused on alterations in the memory phenotype of CD4^+^ and CD8^+^ T lymphocytes after isolation, as well as following CD3/CD28-mediated stimulation and long-term in vitro culture. Moreover, we evaluated the gene expression of key markers associated with T cell activation, effector function, cytokine signaling, and inhibitory regulation.

## 2. Results

### 2.1. Animals

The client-owned group comprises 10 mixed-breed dogs (5 females, including 3 spayed, and 5 males, including one neutered). The mean age and body weight in this group were 4.1 ± 1.7 years and 17.5 ± 5.4 kg, respectively. The short-term stay group consisted of 17 mixed-breed dogs (9 males, 5 of whom were neutered, and 8 females, including 2 that were ovariohysterectomized), with a mean age of 3.2 ± 1.7 years and a mean body weight of 20.9 ± 6.7 kg. The long-term group included 16 mixed-breed dogs (9 males, including 3 neutered, and 7 females, including 4 spayed), with a mean age of 4.1 ± 1.6 years and a mean body weight of 16 ± 6.4 kg.

### 2.2. Hematology and Biochemistry

All blood morphological and biochemical parameters were within reference values [25] as presented in Table 1, with no significant differences between the groups. One long-term stay patient and one client-owned dog had a mild increase in monocyte percentage in blood morphology. Biochemical parameters were within normal limits, with cortisol levels less than 2 μg/dL. Stained blood smears revealed no changes in the cell cytology, other than 10–15% of neutrophils presented with toxic changes observed in 2 short-term stay dogs.

### 2.3. Memory Phenotype

**Figure 1:**
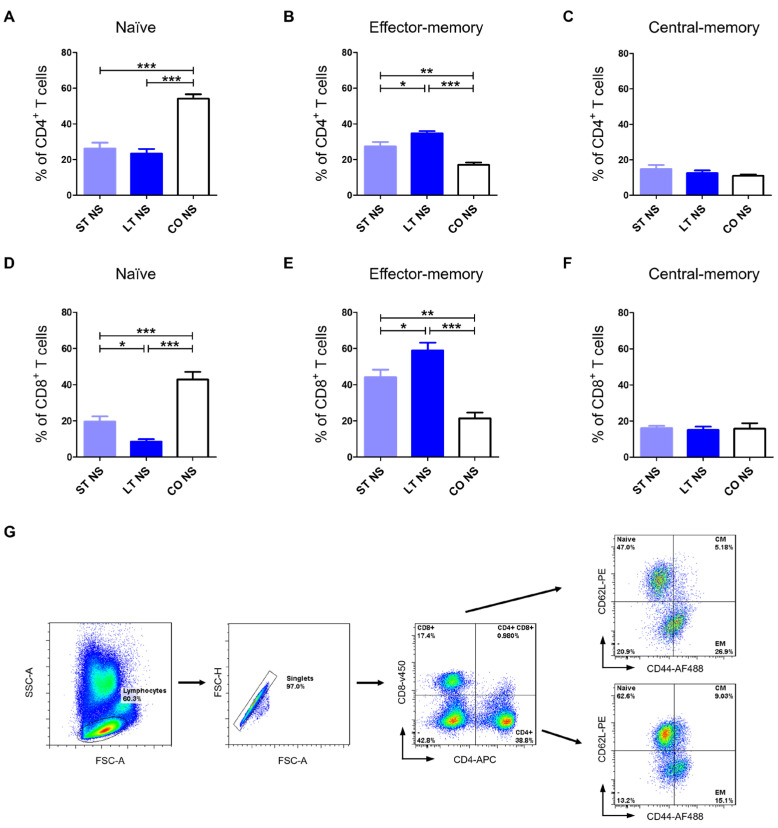
Memory phenotype of lymphocytes analyzed in non-stimulated cells (NS). Bar graphs showing percentage of CD4^+^ T_Naïve_ (**A**), CD4^+^ T_EM_ (**B**), CD4^+^ T_CM_ (**C**), CD8^+^ T_Naïve_ (**D**), CD8^+^ T_EM_ (**E**), and CD8^+^ T_CM_ (**F**) in short-term stay (ST), long-term stay (LT), and client-owned (CO) dogs. Data are shown as the mean results, and error bars indicate SEM. Statistical analysis was performed by one-way analysis of variance (ANOVA) with Tukey’s multiple comparison test (* *p* < 0.05; ** *p* < 0.01; *** *p* < 0.001). (**G**) Gating strategy for flow cytometry analysis. Lymphocytes were gated based on FSC and SSC. Only singlets were included in further analysis. Helper and cytotoxic T cells were distinguished based on CD4 and CD8 expression as presented on a representative cytogram. Among both subsets of lymphocytes, T_Naïve_, T_EM_, and T_CM_ were analyzed based on CD62L and CD44 expression patterns.

Client-owned dogs had significantly the highest percentage of CD4^+^ and CD8^+^ naïve T cells before stimulation (54.09 ± 7.72, 42.77 ± 12.72, respectively) in comparison to the short-term stay group (26.17 ± 11.97, *p* < 0.001; 19.54 ± 10.96, *p* < 0.001) and long-term stay dogs (23.27 ± 10.19, *p* < 0.001; 8.49 ± 4.74, *p* < 0.001). A significantly higher percentage of CD8^+^ naïve T cells was observed in the short-term stay group compared with the long-term stay group (*p* < 0.05) (Figure 1A,D).

Long-term stay dogs had significantly the highest percentage of CD4^+^ and CD8^+^ effector-memory T cells before stimulation (34.67 ± 4.34, 58.94 ± 15.16, respectively) in comparison to the short-term stay group (27.31 ± 9.13, *p* < 0.05; 42.23 ± 14.10, *p* < 0.05) and client-owned stay dogs (17.04 ± 4.05, *p* < 0.001; 21.34 ± 9.12, *p* < 0.001). A significantly higher percentage of CD4^+^ and CD8^+^ effector-memory T cells was observed in the short-term stay group compared with the client-owned stay group (*p* < 0.01) (Figure 1B,E).

There were no differences in CD4^+^ and CD8^+^ central-memory T cells between the shelter and client-owned groups for CD4^+^ T_CM_ (14.61 ± 8.5, 13.09 ± 6.2, 10.98 ± 2.29) and for CD8^+^ T_CM_ (15.08 ± 5.54, 15.46 ± 7.96, 15.70 ± 9.59) (Figure 1C,F).

**Figure 2:**
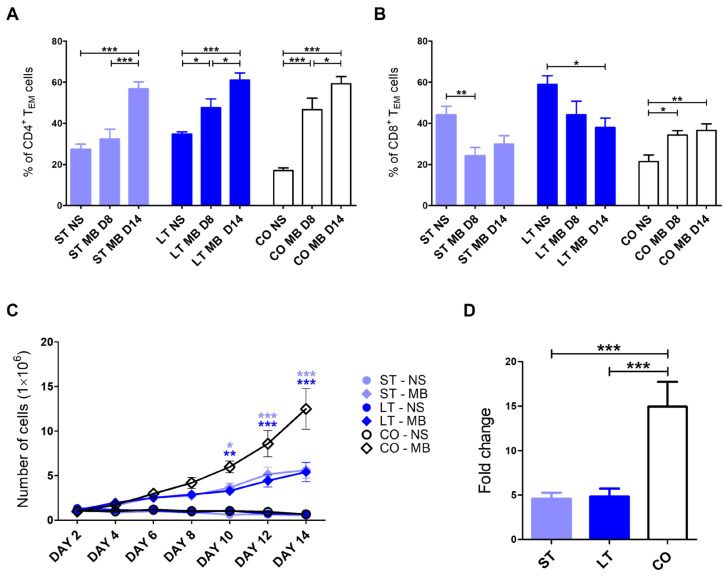
Memory phenotype and expansion of lymphocytes analyzed in non-stimulated cells (NS) and after CD3/CD28 MicroBeads-mediated stimulation (MB) in a 14-day culture. Bar graphs showing the percentage of CD4^+^ T_EM_ (**A**) and CD8^+^ T_EM_ (**B**) in short-term stay (ST), long-term stay (LT), and client-owned (CO) dogs. Data present the mean percentage of non-stimulated cells (NS) and after CD3/CD28 MicroBeads-mediated stimulation in 8-day (MB D8) and 14-day (MB D14) culture. (**C**) Expansion kinetics for canine PBLs cultured for 14 days. (**D**) Bar graph showing fold change in cPBLs number upon activation with MicroBeads on day 14 of culture. Data are shown as the mean results, and error bars indicate SEM. Statistical analysis was performed by one-way analysis of variance (ANOVA) with Tukey’s multiple comparison test (**A**,**B**,**D**) and two-way RM ANOVA with Bonferroni-corrected post hoc comparisons (**C**) (* *p* < 0.05; ** *p* < 0.01; *** *p* < 0.001).

After 14 days post-MicroBeads stimulation, the highest percentage of effector-memory T CD4^+^ cells was observed in shelter and client-owned groups (56.74 ± 13.22, 61.05 ± 11.9, and 59.26 ± 10.75, accordingly) in comparison to the baseline (non-stimulated cells, NS, all *p* < 0.001), and on day 8 (37.87 ± 16.47, *p* < 0.001; 45.45 ± 14.97, *p* < 0.05; 46.68 ± 12.44 *p* < 0.05). Additionally, significantly higher values were observed in long-term stay dogs (*p* < 0.05) and client-owned groups (*p* < 0.001) on day 8 post-stimulation when compared to non-stimulated cells (Figure 2A).

The percentage of CD8^+^ T_EM_ cells was higher in short-term stay dogs before stimulation (44.11 ± 15.10) in comparison with day 8 post-stimulation (24.13 ± 15.80 *p* < 0.01), with no difference on day 14 (29.87 ± 15.93). In long-term stay dogs, there was a difference between the value of CD8^+^ T_EM_ cells measured shortly after blood collection (58.94 ± 15.16, *p* < 0.05) when compared to day 14 (37.95 ± 15.82, *p* < 0.05), with no significant difference on day 8 (44.21 ± 22.61). The CD8^+^ T_EM_ cell percentage in client-owned dogs was significantly lower before stimulation (21.34 ± 9.12) than on day 8 (34.26 ± 4.86, *p* < 0.05) and 14 (36.57 ± 9.97, *p* < 0.01) (Figure 2B).

### 2.4. Expansion of PBLs

PBL numbers were assessed every other day for a total of 14 days of in vitro culture. The expansion kinetics of PBLs revealed a highly significant increase (*p* < 0.001) in cell numbers in the client-owned group starting from day 8 and in the short-term group starting from day 10, and a significant increase (*p* < 0.01) in the long-term shelter group starting from day 10 after CD3/CD28 MicroBeads-mediated activation, compared with non-stimulated cells. From day 12 onwards, changes between MicroBeads-stimulated and non-stimulated cells were highly statistically significant (*p* < 0.001) in all groups. Moreover, statistically significant differences between MicroBeads-stimulated PBLs from shelter dogs (3.67 ± 1.65, *p* < 0.05; 3.3 ± 1.28, *p* < 0.01 for short-term and long-term, respectively) and client-owned dogs (5.88 ± 2.29) were observed from day 10 of the culture period. Lower expansion levels were maintained in both short-term (5.62 ± 3.12, *p* < 0.001) and long-term (5.40 ± 3.72, *p* < 0.001) shelter groups until the end of the culture in comparison with client-owned dogs (12.50 ± 7.9). In addition, lymphocytes isolated from both shelter groups exhibited similar expansion kinetics (Figure 2C). After two weeks of culture, CD3/CD28-stimulated PBLs from client-owned dogs showed a highly significant increase in fold change in cell number (14.94 ± 9.26) compared with both short-term (4.60 ± 2.33) and long-term (4.83 ± 3.06) shelter groups (Figure 2D).

### 2.5. Apoptosis

**Figure 3:**
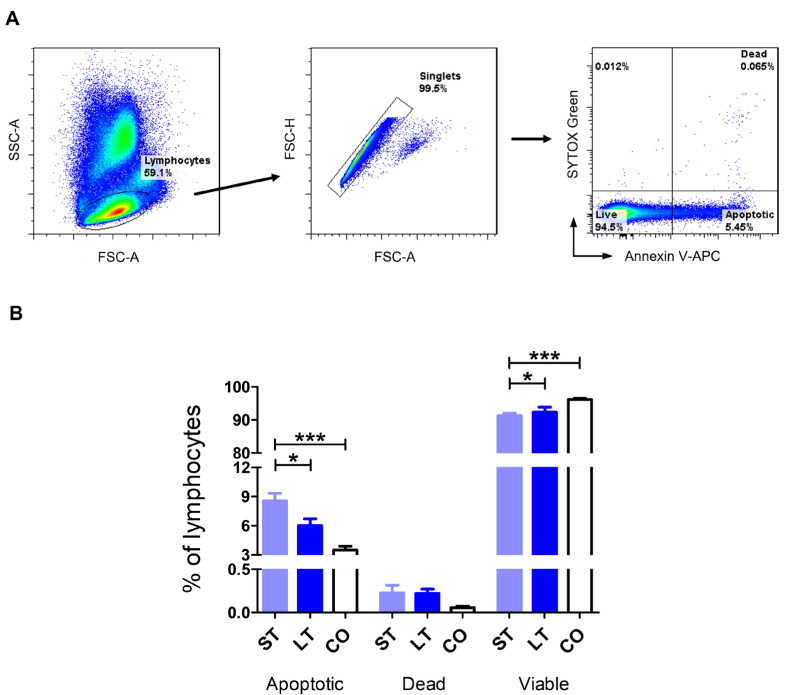
Lymphocyte apoptosis rate. (**A**) Gating strategy for flow cytometry analysis. Lymphocytes were gated based on FSC and SSC. Only singlets were included in further analysis. Apoptotic, dead, and viable cells were distinguished based on Annexin V-APC and SYTOX™ Green staining as presented on representative cytograms. (**B**) Bar graphs showing the percentage of apoptotic, dead, and viable lymphocytes. Data are shown as the mean results, and error bars indicate SEM. Statistical analysis was performed by one-way analysis of variance (ANOVA) with Tukey’s multiple comparison test (* *p* < 0.05; *** *p* < 0.001).

Dogs in the short-term stay group exhibited the highest percentage of apoptosis rate (8.56 ± 3.65), significantly higher than in long-term stay dogs (6.02 ± 2.67, *p* < 0.05) and the client-owned group (3.50 ± 1.12, *p* < 0.001). There were no significant differences between the groups in the percentage of dead cells (0.23 ± 0.42, 0.22 ± 0.2, 0.05 ± 0.04, respectively). Short-term stay dogs had a lower percentage of live cells (91.28 ± 3.59) than long-term stay dogs (92.36 ± 2.6, *p* < 0.05) and client-owned dogs (96.16 ± 1.17, *p* < 0.001) (Figure 3A,B).

### 2.6. Gene Expression

#### 2.6.1. *CD25* and *CD69* Expression

**Figure 4:**
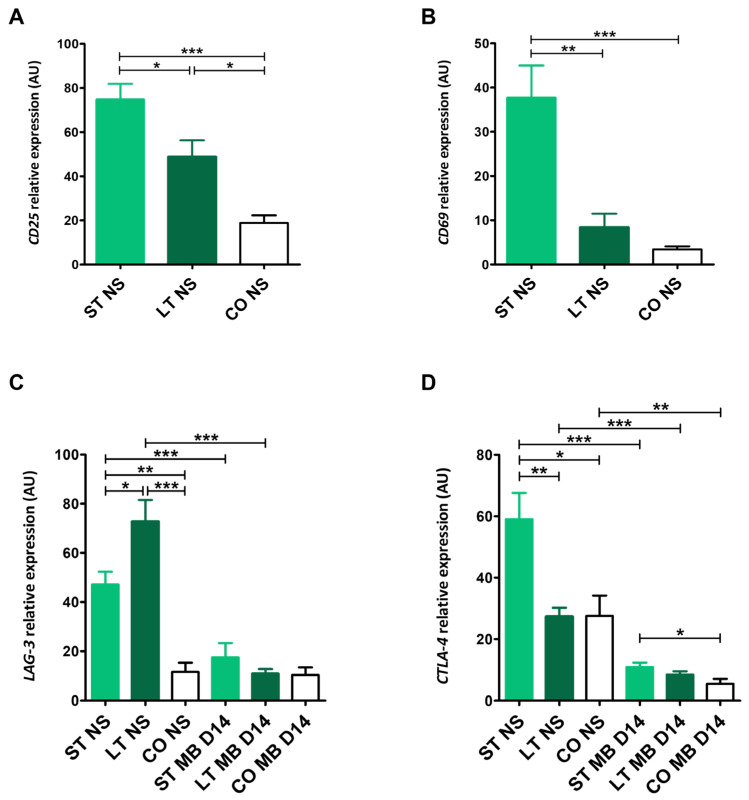
Expression of markers associated with T cell activation and inhibitory regulation. Bar graphs showing relative mRNA expression (AU) of *CD25* (**A**) and *CD69* (**B**) assessed in non-stimulated cells (NS) and *CTLA-4* (**C**) and *LAG-3* (**D**) assessed in non-stimulated cells (NS) and after CD3/CD28 MicroBeads-mediated stimulation (MB) in 14-day culture. Data are shown as the mean results, and error bars indicate SEM. Statistical analysis was performed by one-way analysis of variance (ANOVA) with Tukey’s multiple comparison test (* *p* < 0.05, ** *p* < 0.01, *** *p* < 0.001).

Short-term stay dogs exhibited the highest significant *CD25* expression (74.78 ± 25.6) compared with long-term stay dogs (48.80 ± 21.17, *p* < 0.05) and client-owned dogs (18.91 ± 8.9, *p* < 0.001) (Figure 4A). Similar to *CD69* expression, which was the highest in the short-term group (37.70 ± 25.13) in comparison to other groups (8.37 ± 4.21, *p* < 0.01, and 3.45 ± 1.9, *p* < 0.001, respectively). Long-term stay dogs had significantly higher *CD25* values than client-owned dogs (*p* < 0.05) (Figure 4A,B).

#### 2.6.2. *CTLA-4* and *LAG-3* Expression

Cytotoxic T-lymphocyte antigen 4 (*CTLA-4)* relative expression values shortly after blood collection for short- (58.99 ± 25.72), long-term (27.32 ± 8.58), and client-owned (27.54 ± 13.19) groups were significantly higher than after stimulation (10.89 ± 4.80, *p* < 0.001; 8.44 ± 3.44, *p* < 0.001; 5.43 ± 3.20, *p* < 0.01, respectively) (Figure 4D). Similar differences were found in lymphocyte activation gene 3 (*LAG-3)* expressions, with significantly higher values in short- and long-term groups before (47.19 ± 19.79, 72.74 ± 24.84), with no differences in the client-owed group (11.62 ± 8.34) in comparison to stimulated cells (17.5 ± 21.97, *p* < 0.001; 10.96 ± 5.94, *p* < 0.001; and 10.37 ± 8.68, respectively). The highest *LAG-3* relative expression before stimulation was observed in the long-term group in comparison with the short-term (*p* < 0.05) and client-owned dogs (*p* < 0.001). Also, dogs that stayed in the shelter less than 6 months had a higher *LAG-3* value than client-owned dogs (*p* < 0.01) (Figure 4C). The short-term group had significantly higher *CTLA-4* expression than client-owned dogs before (*p* < 0.05) and after stimulation (*p* < 0.05), and also a higher value shortly after blood collection in comparison with long-term stay ones (*p* < 0.01) (Figure 4D).

#### 2.6.3. *IL-2*, *IFN-γ*, *TNF-α* Expression

**Figure 5:**
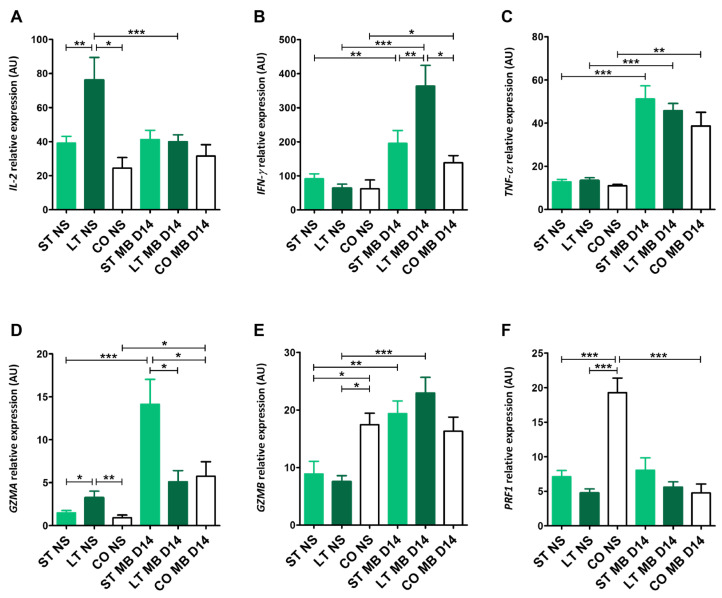
Expression of genes associated with T cell cytokine signaling and effector function. Bar graphs showing relative mRNA expression (AU) of *IL-2* (**A**), *IFN-γ* (**B**), *TNF-α* (**C**), *GZMA* (**D**), *GZMB* (**E**), and *PRF1* (**F**) assessed in non-stimulated cells (NS) and after CD3/CD28 MicroBeads-mediated stimulation (MB) in 14-day culture. Data are shown as the mean results, and error bars indicate SEM. Statistical analysis was performed by one-way analysis of variance (ANOVA) with Tukey’s multiple comparison test (* *p* < 0.05, ** *p* < 0.01, *** *p* < 0.001).

Long-term stay dogs had significantly the highest interleukin-2 (*IL-2*) relative expression (76.14 ± 27.58), higher than short-term (39.14 ± 13.51, *p* < 0.01) and client-owned dogs (24.46 ± 10.79, *p* < 0.05) shortly after blood collection, and significantly higher than after stimulation and 14-day culture (40.01 ± 12.04, *p* < 0.001). After stimulation, relative expression values for short-term stay and client-owned dogs were 41.01 ± 19.86 and 31.52 ± 17.69, respectively (Figure 5A). Interferon-γ (*IFN-γ*) expression values shortly after collection for short-, long-term, and client-owned groups before stimulation did not differ significantly (91.23 ± 55.55, 64.12 ± 38.14, and 61.93 ± 57.62, respectively), and all of them were significantly lower than after stimulation (195.37 ± 113.42, *p* < 0.01; 363.63 ± 160.48, *p* < 0.001; 138.46 ± 59.93, *p* < 0.05, respectively, for each tested group) (Figure 5B). Similar differences were found in tumor necrosis factor alpha (*TNF-α*) expression, with significantly lower values in non-stimulated cells (12.78 ± 4.34, 13.42 ± 4.26, 11.00 ± 1.3) in comparison to post-stimulated cells (51.16 ± 22.00, *p* < 0.001; 45.76 ± 10.10, *p* < 0.001; 38.66 ± 17.90, *p* < 0.01) (Figure 5C). After 14 days post-stimulation, *IFN-γ* expression in long-term stay dogs had a higher value than the other two groups: short-term (*p* < 0.01) and client-owned dogs (*p* < 0.05) (Figure 5B).

#### 2.6.4. *GZMA*, *GZMB*, *PRF1* Expression

Long-term stay dogs had the highest granzyme A (*GZMA*) relative expression (3.25 ± 2.15), significantly higher than short-term (1.49 ± 1.10, *p* < 0.05) and client-owned dogs (0.92 ± 0.81, *p* < 0.01) shortly after blood collection. 14 days post-stimulation, the highest value, 14.1 ± 9.2, was observed in the short-term group. It was significantly higher than the long-term, client-owned groups (5.11 ± 3.89, 5.74 ± 4.49, both *p* < 0.05) and its value before stimulation (*p* < 0.001) (Figure 5D). Granzyme B (*GZMB*) expression showed significantly higher values shortly after blood collection in client-owned dogs (17.45 ± 4.87) than in the short- and long-term groups (8.88 ± 6.58, 7.54 ± 3.46, respectively), both *p* < 0.05. In both shelter groups, *GZMB* expression was also significantly higher 14 days post-stimulation, with values of 19.37 ± 5.81, *p* < 0.01, and 22.95 ± 7.68, *p* < 0.001, respectively. Client-owned dogs had a *GZMB* relative expression value of 16.31 ± 4.87 (Figure 5E). The highest significant expression of perforin 1 (*PRF1*) was in client-owned dogs (19.26 ± 4.7) before stimulation in comparison to shelter-stayed dogs (7.01 ± 3.46, *p* < 0.001 for short-term and 4.74 ± 1.94, *p* < 0.001 for long-term stay dogs). Also, *PRF1* relative expression in client-owned dogs was significantly higher in comparison to 14 days post-stimulation (4.75 ± 3.67, *p* < 0.001). *PRF1* relative expression values were at a similar level after 14-day culture in comparison to non-stimulated cells (8.05 ± 6.6 for short-term and 5.59 ± 2.41 for long-term dogs) (Figure 5F).

## 3. Discussion

Dogs assessed in this study presented no clinical symptoms of stress. They had optimal BCS and no demeanor issues, such as obsessive-compulsive disorder symptoms. All dogs included in the study have regular contact and interactions with humans (walks, socialization, etc.). These findings were similar to our previous ones described in Kulka et al. 2025 [7]. Laboratory results further supported the observations of animal welfare: low cortisol levels, no sign of a stress leukogram, and a stable neutrophil/lymphocyte ratio. Additionally, cytology evaluation revealed no signs of moderate or marked lymphocyte activation. However, our research showed changes on the subcellular level. We investigated the memory profile of T lymphocytes by classifying canine CD4^+^ and CD8^+^ T cells into distinct subsets according to their migratory pattern. Expression patterns of CD44 and CD62L molecules [19] can be used to identify specific canine T cell subpopulations, including naïve T cells (T_naïve_, CD44^low^CD62L^high^), central-memory T cells (T_CM_, CD44^high^CD62L^high^), and effector-memory T cells (T_EM_, CD44^high^CD62L^low^) [26]. In the present study, we observed significant differences in the proportions of T_naïve_ and T_EM_ cells within both helper and cytotoxic T lymphocyte subsets. Client-owned dogs had a significantly higher percentage of naïve CD4^+^ and CD8^+^ T lymphocytes when compared to both short-term stay and long-term stay groups (*p* < 0.001). Such changes may reflect reduced antigenic stimulation associated with environmental factors, as antigen exposure is a key driver of naïve T cell activation and differentiation into effector and memory subsets [27,28,29]. Additionally, in the CD8^+^ T cell subset, the percentage of naïve T cells was lower in long-term stay dogs when compared with short-term stay (*p* < 0.05), possibly due to prolonged environmental antigen exposure [30,31]. Our results showed an opposite change in effector-memory T cells, with the highest values in long-term stay dogs, suggesting an ongoing immune response with similar involvement of CD8^+^ and CD4^+^ T cells. Short-term stay dogs have lower reactivity than the long-term stay group (*p* < 0.05), but still significantly higher than the client-owned animals (*p* < 0.01). No changes were seen in the percentage of central-memory T cells, which reflects resembling response capacity in all tested groups.

There is currently no available data addressing the impact of chronic stress on immunomodulation and hematological parameters in shelter dogs. Existing studies have primarily focused on behavioral assessments [32,33,34,35] or, to a limited extent, cortisol measurements [36,37,38]. However, our previous research indicates that stress-induced changes at the cellular and molecular levels are highly relevant [7]. Sustained antigenic stimulation is known to drive progressive T cell differentiation from naïve toward effector and memory phenotypes, as described in classic models of T cell responses, where antigen exposure promotes effector expansion and subsequent memory formation during prolonged infections or immune activation [39,40,41]. Studies revealed that dogs exhibit a decreased percentage of naïve CD4^+^ and CD8^+^ T cells and increased terminal effector CD8^+^ T cells in response to chronic stress exposure [42,43]. To date, investigations on canine T cells have been conducted almost exclusively in the context of diseases with an inflammatory component or age-related immune changes. Bauman et al. [42] demonstrated that canine peripheral blood contains distinct naïve and memory T lymphocyte populations and that these proportions shift under physiological stressors. These results are in line with our findings of reduced naïve CD4^+^ and CD8^+^ T cells in long-term stay shelter dogs, suggesting that prolonged environmental antigen exposure may parallel aspects of time-related differentiation in canine T cell populations. Furthermore, they are consistent with findings in humans and mice [29,42]. Measurements of T cell subset differences in peripheral blood are considered to be helpful in exploring systemic immune responses in clinical patients [44,45,46,47]. Also, in canines with dermatologic inflammation, alterations of T cell subpopulations proved the utility of this approach to characterize immune responses in disease processes [42,48].

Additionally, in our investigation, we evaluated T cell response to in vitro stimulation mediated through CD3 and CD28 molecules in different groups. Client-owned dogs presented an increase in the percentage of effector-memory T cells after 8 and 14 days. The CD8^+^ T lymphocyte population acquired the effector-memory phenotype by day 8 and maintained it until day 14 of in vitro culture. In contrast, CD4^+^ T cells exhibited a gradual increase in this phenotype throughout the 14-day period. A gradual increase was also observed in the long-term stay group of shelter dogs, with the highest levels of effector-memory CD4^+^ T cells recorded on the final day of culture. In contrast, short-term stay dogs showed no significant changes until day 8, indicating a more delayed differentiation response. A decreasing pattern was observed in CD8^+^ T cells from both groups of shelter dogs. Short-term stay dogs (up to 6 months) responded more rapidly, showing significant changes by day 8, whereas long-term stay dogs exhibited lower levels on day 14 of in vitro culture. This pattern contrasts with client-owned dogs, in which the lowest percentages were observed at the beginning of stimulation and increased over time. The observed decrease in effector T cell proportions in both groups of shelter dogs may reflect immune overstimulation, resulting from the additive effect of an already elevated baseline level of these cells, combined with the additional activating signal provided in vitro [49]. Only a few studies address the changes in canine T cell memory phenotypes after stimulation. Activation of canine CD4^+^ T lymphocytes via anti-CD3/CD28 magnetic beads promotes expansion of effector-memory T cells, accompanied by IL-2 and IFN-γ production [26]. Lang et al. [50] demonstrated that rabies vaccination promoted effector-memory and terminally differentiated effector-memory phenotype in CD4^+^ T cells in blood and spleen. Additionally, stimulation with plant mitogen, concanavalin A, also induces a shift in memory subset proportions, supporting the utility of memory phenotyping as a functional readout of canine T cell responses [26,43]. Moreover, age has been shown to influence the dynamics of memory subsets upon stimulation, indicating that the baseline distribution of naïve T cells may determine the trajectory of memory differentiation [51].

The results of the present study demonstrate that canine PBLs respond robustly to CD3/CD28 MicroBeads-mediated stimulation, showing progressive expansion during prolonged in vitro culture. However, the kinetics of this response differed markedly between groups. Similarly to previous studies on canine lymphocytes, CD3/CD28 stimulation effectively induced T cell proliferation, confirming the suitability of this activation method for canine immune cells [26]. Earlier reports have shown that canine peripheral blood lymphocytes typically exhibit significant expansion between days 7 and 10 of culture following stimulation, which is consistent with the early and highly significant increase observed in the client-owned group in the present study [26,52]. In contrast, lymphocytes derived from shelter dogs displayed delayed and reduced expansion capacity, with significant increases occurring later and remaining at lower levels throughout the culture period. Comparable impairments in lymphocyte proliferation have been described in dogs with altered immunological status, such as older animals or populations with chronic immune challenges [51]. That suggests that long-term stress may negatively affect T cell responsiveness and proliferative potential. Notably, the similar expansion kinetics observed in both short-term and long-term shelter groups may indicate that even relatively brief shelter exposure is sufficient to alter lymphocyte expansion capacity. These findings support previous observations that physiological and environmental factors strongly influence canine lymphocyte function and highlight the importance of housing conditions.

Chronic stress has been shown to promote apoptosis of lymphocytes, contributing to immunosuppression. Studies demonstrated that prolonged physical restraint increases Fas (CD95)-mediated apoptosis of lymphocytes in animal models, implicating programmed cell death pathways in stress-induced lymphocyte loss [53,54]. Therefore, the next step of our study was to assess the apoptotic rate of canine T lymphocytes. We revealed that short-term stay dogs had the lowest percentage of viable cells and the highest percentage of apoptotic cells. These differences were statistically significant in comparison to both long-term stay and client-owned dogs. That may indicate short-term dog T cells are more prone to stress-related apoptosis in comparison to long-term ones, which may develop better adjustment mechanisms to prolonged stress exposure. These data revealed a pattern similar to that observed in our previous study on neutrophil and monocyte apoptosis rates [7], indicating that stress-related immune modulation has a universal impact on different types of leukocytes. Studies investigating the impact of stress on lymphocyte apoptosis have not been conducted in dogs. However, interesting data have been obtained from studies in rats exposed to stressful conditions, which were reflected by an increased apoptotic index in PBMCs. Importantly, this effect was reversible, with the apoptotic index returning to control levels after a 2- or 4-week recovery period [55]. These observations are consistent with our findings in long-term stay dogs. Given the absence of significant differences in lymphocyte apoptosis between long-term stay and client-owned dogs, it is possible that shelter dogs develop adaptive mechanisms in response to persistent environmental stress. Similarly, in humans, psychological stress correlates with increased T cell apoptosis in vitro, suggesting that stress-associated apoptosis is not limited to animal models [56].

Investigation of the transcriptional status of lymphocytes in dogs exposed to shelter-associated stress was performed by gene expression analysis encompassing markers of early T lymphocyte activation, cytokine signaling, cytotoxic effector function, and inhibitory immune regulation. This approach allowed us to assess how prolonged stress shapes immune response at the transcriptional level directly after blood collection and following long-term in vitro activation.

Firstly, we focused on genes associated with early T lymphocyte activation, as their expression reflects the baseline immunological status and provides essential context for the interpretation of downstream effector and regulatory pathways [52]. *CD25* (α-chain of IL-2 receptor) and *CD69* (C-type lectin) exhibited distinct expression patterns in all tested groups. *CD69* expression was markedly elevated in the short-term stay group, reaching levels significantly higher than those observed in both long-term stay and client-owned dogs. In contrast, *CD69* expression in long-term shelter dogs did not differ significantly from that of control dogs. Given that *CD69* is rapidly induced following T lymphocyte activation and is commonly associated with immediate early responses to stress-related signaling, its selective upregulation in short-term stay dogs suggests recent or ongoing immune activation triggered by acute environmental stress [57,58,59]. A similar but more graded pattern was observed for *CD25* expression, with significant differences between all tested groups, with the highest values in short-term stay dogs. The progressive reduction in *CD25* expression with increasing duration of shelter stay suggests attenuation of this activation signal over time, likely reflecting the engagement of regulatory mechanisms limiting sustained immune stimulation [60,61]. Prolonged immune activation can be counterbalanced by regulatory pathways that dampen expression of activation markers to prevent immune overactivation or exhaustion [62].

Importantly, the pronounced expression of early activation markers in short-term stay dogs was accompanied by reduced expression of cytotoxic effector genes such as *GZMB* and *PRF1* and increased expression of inhibitory receptors *CTLA-4* and *LAG-3*. Perforin and granzyme B together constitute the major enzymes released by cytotoxic T lymphocytes and natural killer (NK) cells, which induce apoptosis of target cells [63,64,65]. Downregulation of *GZMB* and *PRF1* expression, therefore, reflects diminished cytotoxic competence rather than reduced immune cell numbers. This discrepancy between early activation signals and effector function indicates that stress induces partial T lymphocyte activation and diminishes the effectiveness of cytotoxic differentiation. T cell activation appears to be affected by inhibitory pathways, resulting in a regulated and functionally restrained immune state. These findings are consistent with the literature demonstrating that stress and stress-related hormone signaling can impair effector functions of cytotoxic T lymphocytes while simultaneously promoting expression of inhibitory receptors associated with immune exhaustion. For example, chronic stress in experimental models has been shown to induce upregulation of inhibitory receptors such as LAG-3 on CD8^+^ T cells, together with suppressed secretion of effector molecules like granzyme B [66]. The inhibitory receptors CTLA-4 and LAG-3 serve as immune checkpoints that down-modulate T cell activation and are commonly associated with restrained cytotoxic activity [67,68]. Increased expression of such receptors has been shown to correlate with reduced effector function, for example, leading to lower expression of perforin and granzymes in both T cells and NK cells that express these markers. That may indicate the presence of an immune regulatory or suppressed phenotype rather than an active, fully cytotoxic state [69]. In long-term shelter dogs, lower expression of both early activation markers (*CD69*, *CD25*) suggests immunomodulation and establishment of a more stable, stress-adapted immune phenotype. These observations are supported by the concurrent upregulation of inhibitory receptors, especially *LAG-3,* and alterations in cytotoxic gene expression observed in this group. Such regulatory adaptation has been observed in other models of chronic immune stimulation and stress, where prolonged exposure leads to a stable yet functionally limited immune state characterized by high inhibitory receptor expression and dampened cytotoxic effect [69]. By contrast, client-owned dogs exhibited low baseline expression of both early activation and inhibitory markers. This is consistent with a resting immune state characterized by preserved cytotoxic competence and absence of stress-induced activation.

Granzyme A (*GZMA*) displayed a different expression pattern from granzyme B. The baseline expression level of *GZMA* was significantly higher in long-term stay dogs when compared to both short-term stay (*p* < 0.05) and client-owned dogs (*p* < 0.01), while expression in short-term stay and client-owned dogs was lower and comparable. Granzyme A, although originally characterized as a component of cytotoxic T lymphocyte granules alongside perforin and granzyme B, has been increasingly recognized as a marker of chronic immune activation, low-grade inflammation, and stress-associated immune dysregulation rather than acute cytotoxic effector function [70,71]. Its upregulation in long-term shelter dogs may indicate an alteration in cytotoxic T lymphocyte transcriptional activity, potentially associated with adaptation to chronic environmental stress. In contrast, the lower baseline *GZMA* expression in short-term stay dogs might reflect acute activation that has not yet resulted in stable transcriptional remodeling. Overall, the distinct pattern of *GZMA* expression in comparison to *GZMB* and *PRF1* suggests that chronic environmental stress experienced during prolonged shelter stay may drive changes in cytotoxic T lymphocyte transcriptional activity that differ from acute activation, potentially reflecting immune modulation toward inflammation-associated roles rather than classic cytotoxic effector function.

Cytokine expression patterns further provide support for the concept of differential immune adaptation under stress. *IL-2*, a crucial growth and survival factor for T lymphocytes, was significantly elevated in long-term stay dogs in comparison to short-term stay and client-owned animals. IL-2 plays well-established roles in promoting T cell proliferation and survival, and is also involved in the differentiation and maintenance of regulatory T cells under chronic stimulatory conditions [61,72,73]. Elevated *IL-2* in long-term stay dogs, occurring together with suppressed cytotoxic gene expression, may reflect a compensatory mechanism supporting T lymphocyte viability and immune balance under chronic stress conditions. This concept aligns with evidence from an experimental mouse model in which IL-2 can contribute to T cell maintenance and regulatory functions distinct from classical pro-inflammatory cytotoxic pathways [74,75,76]. *IFN-γ* expression, however, remained low and comparable across all groups, indicating that shelter-associated stress did not trigger Th1 polarization or overall inflammatory activation in peripheral lymphocytes. This pattern contrasts with experimental or disease models in dogs, where *IFN-γ* and *IL-2* are often upregulated under strong immune stimulation, such as infection or tumor regression [77,78]. The absence of *IFN-γ* upregulation in our study suggests that shelter-associated stress primarily triggers pathways supporting T cell maintenance and survival (reflected by *IL-2* upregulation), rather than pro-inflammatory Th1-type activation [79]. Together, these findings suggest that chronic stress promotes selective adjustments in T lymphocyte transcriptional activity, favoring pathways associated with stress adaptation and cellular maintenance, without inducing broad inflammatory responses.

Taken together, the profiles of early activation markers, cytotoxic enzymes, inhibitory receptors, and cytokines indicate that T lymphocyte adaptation to shelter stress is dynamic and context-dependent. Short-term exposure predominantly triggers acute activation and early inhibitory signaling, whereas long-term exposure results in a stabilized, stress-adapted immune state, characterized by sustained *GZMA* expression and enhanced IL-2-mediated homeostatic support.

Following 14 days of in vitro culture after CD3/CD28 stimulation, T lymphocyte transcriptional profiles exhibited distinct, gene-specific dynamics across the groups, indicating differential capacities for transcriptional adaptation depending on prior stress exposure. This observation underscores that not only baseline transcriptional states but also in vitro responsiveness can be shaped by in vivo experiences, such as chronic environmental stress. Previous studies have demonstrated that activation of canine T lymphocytes using anti-CD3/CD28 stimulation leads to robust activation and induction of effector functions, including cytokine production and proliferation. Canine peripheral blood lymphocytes isolated from healthy donors and stimulated in vitro exhibit effective activation and expansion accompanied by increased IL-2 and IFN-γ secretion [26,80]. We revealed that among cytotoxic effector genes, *GZMB* expression increased significantly in both short-term (*p* < 0.01) and long-term stay dogs (*p* < 0.001) after in vitro stimulation, whereas no significant change was observed in client-owned dogs. By day 14, *GZMB* expression levels were comparable across all groups, suggesting that cytotoxic potential can be enhanced under in vitro activation and culture conditions. The enhanced induction of *GZMB* in shelter dogs may reflect increased transcriptional responsiveness of cytotoxic effector pathways during prolonged in vitro culture. In contrast, the absence of a similar increase in client-owned dogs suggests that cytotoxic effector gene expression may already be maintained at a relatively stable baseline level and therefore shows limited additional transcriptional induction.

*PRF1* expression followed yet another trajectory during in vitro culture. In client-owned dogs, initially higher baseline *PRF1* expression declined significantly over the 14-day culture, whereas expression remained stable in both shelter groups. As a result, *PRF1* expression reached comparable levels across all groups by day 14. This pattern suggests that baseline differences in perforin expression are not fixed and can be modulated by prolonged culture conditions. The decline observed in client-owned dogs contrasts with the relative stability observed in shelter dogs, indicating differences in the regulation of perforin expression during in vitro activation and culture. At present, direct comparison of these findings with previously published studies in canine lymphocytes is challenging, as available canine immunology literature does not include analyses of *PRF1* transcriptional dynamics, especially during prolonged in vitro culture following stimulation. Most studies in dogs have focused on cytokine production, proliferation, or phenotypic characterization of lymphocyte subsets, rather than long-term assessment of cytotoxic effector gene regulation. Consequently, our observations provide novel insight into perforin regulation in canine T lymphocytes under defined in vitro activation conditions. Nevertheless, relevant information regarding regulatory mechanisms can be derived from human and murine studies, in which perforin regulation is known to involve tightly controlled transcriptional and post-transcriptional mechanisms. In both humans and mice, *PRF1* is subject to complex transcriptional control during the differentiation of cytotoxic T lymphocytes and natural killer cells, and its expression can be maintained or suppressed depending on cytokine milieu, activation signals, and the differentiation state of the cell [81,82]. Within this context, the results obtained in the present study are consistent with the possibility that *PRF1* expression in canine T lymphocytes may also be influenced by factors related to activation and differentiation status. However, the current study does not address the underlying regulatory mechanisms directly. Given the limited number of studies on perforin regulation in dogs, particularly in the context of prolonged in vitro culture and stimulation, these findings highlight a gap that needs further investigation to better understand how cytotoxic effector genes are regulated in the canine immune system.

Cytokine gene expression further underscored group-specific adaptive responses. In long-term stay dogs, *IL-2* expression declined significantly by day 14, whereas it remained unchanged in short-term stay and client-owned dogs, leading to comparable *IL-2* levels across all groups at the end of culture. This reduction in long-term stay dogs may reflect diminishing reliance on IL-2-mediated homeostatic support once cells are removed from the chronic stress environment and maintained under uniform culture conditions. Previous studies in dogs have demonstrated that *IL-2* is inducible upon activation of peripheral blood mononuclear cells, and its expression is associated with T cell maintenance and early activation rather than terminal effector differentiation [42,83]. *IFN-γ* expression increased significantly in all groups, indicating that prolonged stimulation is associated with the acquisition of effector-associated transcriptional features in canine T lymphocytes. This aligns with previous reports showing that canine PBMC or T cells upregulate *IFN-γ* mRNA after in vitro activation, either by mitogen stimulation or antigen-specific exposure [19,26,84,85]. Notably, long-term stay dogs exhibited the highest *IFN-γ* expression at day 14, suggesting that chronic stress exposure may prime lymphocytes for enhanced *IFN-γ* responsiveness upon activation, even though baseline expression remained low. *TNF-α* expression showed a uniform pattern across all groups, with significant upregulation during in vitro culture from initially low baseline levels to comparable expression at day 14. This suggests that *TNF-α* induction reflects generalized activation-associated transcriptional remodeling under prolonged culture conditions, rather than stress-specific regulation. Such patterns are consistent with prior studies in dogs showing robust induction of pro-inflammatory cytokines following T cell activation in vitro [86].

Inhibitory receptors exhibited a downregulation during prolonged in vitro culture. Both *LAG-3* and *CTLA-4* expression decreased significantly over the 14-day culture period, indicating that stress-associated inhibitory signaling observed at baseline is not sustained under extended in vitro conditions. *LAG-3* expression declined significantly in both short- and long-term stay dogs, reaching levels similar to client-owned dogs, whose expression remained stable throughout the culture period. Similarly, *CTLA-4* expression decreased significantly in all groups relative to baseline, although short-term stay dogs retained higher expression levels than client-owned animals at day 14. Current knowledge regarding *LAG-3* and *CTLA-4* expression in canine T lymphocytes during prolonged in vitro culture remains limited. Previous studies in dogs have demonstrated that inhibitory receptors, including *CTLA-4*, are expressed on T cells in chronic inflammatory or neoplastic conditions, highlighting their functional relevance in the canine immune system [87,88,89]. However, detailed kinetics of *LAG-3* and *CTLA-4* expression in canine T cells under extended culture conditions remain largely unexplored. In human and murine T cells, *CTLA-4* and *LAG-3* are rapidly induced upon T cell activation through TCR and costimulatory signals, acting as key modulators of effector responses and regulatory functions [90,91]. Importantly, their expression is dynamic: *CTLA-4* gene expression and surface protein levels increase following T cell activation but decrease as cells enter a quiescent state or receive sustained IL-2 stimulation without continued TCR engagement [92,93,94]. Similarly, *LAG-3* is transiently upregulated upon activation and downregulated as cells differentiate and activating stimuli diminish [95,96]. These observations are consistent with our findings that prolonged culture with IL-2, following initial activation, is not sufficient to maintain elevated inhibitory receptor expression. Although inhibitory receptors such as *LAG-3* and *CTLA-4* were induced at baseline in association with stress and partial T lymphocyte activation, their expression decreased over time during in vitro culture, indicating that stress-associated inhibitory transcriptional regulation is dynamic and reversible. The overall reduction in *LAG-3* and *CTLA-4* across all groups suggests that sustained IL-2 signaling alone does not preserve heightened inhibitory receptor expression in the absence of continued activating stimuli. Notably, *CTLA-4* expression remained higher in short-term stay dogs at day 14, which may reflect remaining regulatory characteristics from recent acute stress exposure. However, the overall downward trend across all groups highlights the reversible nature of stress-associated inhibitory transcriptional regulation.

In conclusion, our study highlights the need to develop new and more sensitive methods for assessing canine welfare, particularly those capable of detecting subtle cellular and molecular alterations. We demonstrate that T lymphocytes isolated from shelter dogs differ markedly in their distribution of naïve and effector-memory subsets compared with client-owned dogs. These differences persist during CD3/CD28 MicroBeads-mediated activation and extended in vitro culture, suggesting that different durations of stress and environmental antigen exposure distinctly shape immune responsiveness. Although PBLs from shelter dogs retained the ability to proliferate upon activation, their expansion capacity was significantly reduced relative to that observed in client-owned dogs. Furthermore, shelter-associated stress was linked to increased lymphocyte apoptosis, indicating compromised immune cell resilience. Gene expression analysis revealed both reversible and persistent components of stress-induced immune modulation. Although many baseline transcriptional differences between the groups diminished after prolonged culture, several important differences remained evident. Importantly, this study is the first to identify several key distinctions, including stable *GZMA* expression in long-term stay dogs and heightened transcriptional plasticity in short-term stay dogs.

These results support our concept that environmental stress shapes not only the initial transcriptional profile of T lymphocytes but also their capacity for subsequent adaptation. Different durations of stress exert qualitatively distinct effects on immune transcriptional alterations, underscoring the complex and dynamic nature of stress-mediated immune modulation in dogs.

## 4. Materials and Methods

### 4.1. Animals and Material Collection

The first group included 33 healthy, mixed-breed dogs with a Body Condition Score (BCS) of 4–5 on a 9-point scale, indicating an optimal body condition [97,98,99]. All dogs were housed in the same shelter under uniform environmental conditions, which included regular daily walks and human interaction. They were fed a similar nutritionally balanced diet and had free access to water. The dogs were between 2 and 6 years old. Efforts were made to ensure the group was as uniform as possible in terms of BCS, body weight, and age. Shelter dogs were further subdivided into two groups based on the duration of their stay. The short-term group (ST, referred to as short-term stay) included 17 dogs (9 males, 8 females) that had resided in the shelter for up to 6 months. The long-term group (LT, referred to as long-term stay) comprised 16 dogs (9 males, 7 females) with a length of stay ranging from 6 months to 2 years. This classification approach is consistent with our previous study [7]. The second group comprised 10 clinically healthy, client-owned (CO) dogs (5 males, 5 females), aged between 1.5 years and 6 years and 4 months, all with a Body Condition Score (BCS) of 4–5. These dogs lived exclusively in domestic home environments. All dogs were routinely vaccinated and received antiparasitic prophylaxis. None had received any medications prior to blood collection, and none belonged to breeds predisposed to immunodeficiency syndromes. All females were in anestrus with no confirmed pregnancy. Each dog underwent a clinical examination performed by a licensed veterinary surgeon, and blood samples were collected during routine health checkups following the acquisition of written informed consent from the owner. Research was conducted in the Republic of Poland. Animals with any diseases or undergoing treatment were excluded from analysis. Blood acquisition was performed for the first time in the morning, within the same time window for all dogs, following a 12 h fasting period, and under conditions designed to minimize stress. Blood samples were drawn from the cephalic vein and collected into EDTA-K3 anticoagulant tubes and clotting tubes (both supplied by FL Medical, Torreglia, Italy). Serum was separated by centrifugation of the clotted blood samples and subsequently aspirated for analysis.

### 4.2. Hematological and Biochemical Analysis

Whole blood samples were analyzed for Complete Blood Count (CBC) using the Sysmex XN-1000V Series Hematology Analyzer (Sysmex Europe SE, Norderstedt, Germany). Serum samples were evaluated for biochemical parameters, including alanine aminotransferase (ALT), aspartate aminotransferase (AST), alkaline phosphatase (ALP), urea, creatinine, total protein (TP), albumin, globulin, lipase, cortisol, and C-reactive protein (CRP). All biochemical assays were conducted on the Roche Cobas C501 Chemistry Analyzer (Roche Diagnostics, Rotkreuz, Switzerland). Additionally, blood smears were prepared, stained with May-Grünwald-Giemsa stain according to manufacturer instructions, and examined microscopically.

### 4.3. Canine Peripheral Blood Lymphocytes (PBLs) Isolation

Protocols for the isolation, culture, and extracellular staining of canine peripheral blood mononuclear cells (PBMCs) are well established in our laboratory [7,26,100]. Collected whole blood samples were diluted with sterile phosphate-buffered saline (PBS, Sigma-Aldrich, Taufkirchen, Germany) at a 1:2 ratio and carefully layered onto a density gradient medium (Lymphoprep, Stemcell Technologies, Vancouver, BC, Canada). Density gradient centrifugation was performed using SepMate PBMC isolation tubes (Stemcell Technologies, Vancouver, BC, Canada) at 800× *g* for 10 min at room temperature (RT). Next, the isolated cells were washed twice with PBS supplemented with 2 mM EDTA (Sigma-Aldrich, Taufkirchen, Germany) and 2% FBS (Thermo Fisher Scientific, Waltham, MA, USA). Remaining erythrocytes were eliminated by a 4 min incubation with ACK lysing buffer (Thermo Fisher Scientific, Waltham, MA, USA) at room temperature. After this procedure, the cells were washed with sterile PBS and enumerated using an automated cell counter (Countess II Automated Cell Counter, Thermo Fisher Scientific, Waltham, MA, USA). Cell viability was evaluated by staining with 4% Trypan blue (Thermo Fisher Scientific, Waltham, MA, USA). The isolated cells were resuspended in a culture medium consisting of RPMI-1640 GlutaMAX™ medium supplemented with 10% FBS, 1% sodium pyruvate, 1% nonessential amino acids, 0.1% HEPES, and 1% antibiotics: penicillin and streptomycin (all from Thermo Fisher Scientific, Waltham, MA, USA). PBMCs were incubated overnight at 38.5 °C, 5% CO_2_ (Sanyo Electric., Ltd., Osaka, Japan) at a concentration of 2.5 × 10^6^ cells/mL in 6-well plates (Corning, New York, NY, USA) for monocyte/macrophage depletion through plastic adherence. On the following day, non-adherent canine PBLs were collected and counted. PBLs were plated at a density of 2 × 10^6^ cells/mL and stimulated using nano-sized magnetic beads (termed as MicroBeads, Miltenyi Biotec, Bergisch Gladbach, Germany) coated with cross-linking anti-canine CD3 antibody (clone CA17.2A12, Bio-Rad, Hercules, CA, USA) and anti-canine CD28 antibody (clone 1C6, Functional Grade, eBioscience, Thermo Fisher Scientific, Waltham, MA, USA) at the concentrations recommended by the manufacturer. After 24 h of activation, MicroBeads were removed, and the medium was supplemented with 10 ng/mL of recombinant canine IL-2 (R&D Systems, Minneapolis, MN, USA). PBLs were cultured for 14 days, enumerated every 2 days, and provided with fresh IL-2-supplemented medium throughout the culture period. Cells were harvested on the days indicated and used for flow cytometry analysis and real-time PCR analysis. All experiments included a minimum of 10 biological replicates per animal group.

### 4.4. Extracellular Staining

Extracellular staining was performed directly after PBMCs isolation after 8 and 14 days of in vitro culture. Cells were washed twice and resuspended in 100 µL of sterile FACS buffer (PBS supplemented with 2% FBS). Fc Receptor Binding Inhibitor (eBioscience, Thermo Fisher Scientific, Waltham, MA, USA) was applied for 20 min to reduce non-specific binding. To distinguish helper and cytotoxic T lymphocytes, cells were stained with anti-human CD4-APC (clone YKIX302.9) and anti-canine CD8a-v450 (clone YCATE55.9) (eBioscience, Thermo Fisher Scientific, Waltham, MA, USA). Additionally, to assess memory phenotype, rat anti-canine CD44-Alexa Fluor 488 (clone YKIX337.8.7) and mouse anti-human CD62L-PE (clone FMC46) antibodies (Bio-Rad, Hercules, CA, USA) were used. Following antibody incubation, the cells were washed twice with FACS buffer, centrifuged at 300× *g* for 4 min, and resuspended in 200 µL of FACS buffer for subsequent analysis.

### 4.5. Apoptosis Assay

Apoptosis levels were evaluated directly after PBMCs isolation using the Dead Cell Apoptosis Kit with Annexin V-APC and SYTOX™ Green (Invitrogen, Thermo Fisher Scientific, Waltham, MA, USA). The apoptosis assay protocol for canine PBMCs was optimized in our laboratory through prior validation and testing [100]. Cells were washed with 1× annexin-binding buffer and resuspended at a concentration of 1.25 × 10^6^ cells/mL. Staining was performed according to the manufacturer’s instructions with 5 μL of Annexin V-APC and 1 μL of the 1 μM SYTOX™ Green working solution added to every 100 μL of cell suspension. Cells were incubated at 37 °C with 5% CO_2_ for 15 min. Next, 100 μL of 1× annexin-binding buffer was added, and samples were analyzed immediately after staining.

### 4.6. Flow Cytometry Analysis

Flow cytometry analyses were conducted using a BD FACS Aria II flow cytometer (Becton Dickinson, Heidelberg, Germany). Each time, a minimum of 50 000 events were recorded. The obtained data were analyzed with FlowJo 7.6.1 software (TreeStar Inc., Ashland, OR, USA). Lymphocytes were gated based on their size and granularity using forward-scatter (FSC) and side-scatter (SSC) parameters. Only singlets were included into analysis.

### 4.7. RNA Isolation, cDNA Synthesis, and Real-Time PCR Analysis

Cells were collected after isolation and again after 14 days of in vitro culture subsequent to CD3/CD28-mediated activation. Cells were rinsed with sterile PBS and centrifuged at 300× *g* for 8 min. The resulting cell pellets were resuspended in 100 µL of lysis buffer, and total RNA was extracted using the RNAqueous™-Micro Total RNA Isolation Kit (Invitrogen, Thermo Fisher Scientific, Waltham, MA, USA) in accordance with the manufacturer’s protocol. DNase treatment was applied to eliminate genomic DNA, and RNA yield was quantified using a NanoDrop 2000 spectrophotometer (NanoDrop Technologies, Wilmington, DE, USA). Reverse transcription was performed on 1 µg of total RNA using the High-Capacity FG RNA-to-cDNA Kit (Applied Biosystems, Thermo Fisher Scientific, Waltham, MA, USA). Quantitative real-time PCR analysis was carried out with SYBR Green Select Master Mix (Applied Biosystems, Thermo Fisher Scientific, Waltham, MA, USA) following the manufacturer’s guidelines on an AriaMx Real-Time PCR System (Agilent Technologies, Santa Clara, CA, USA). The primers for *CD69* and *CD25* were designed and used in our previous study [26]. The other primers used in our study were reported previously by Park et al. (*IFN-γ*) [101], Tani et al. (*IL-2*, *TNF-α*) [102], Temizkan et al. (*GZMA*, *GZMB*, *PRF1*) [103], de Souza et al. (*LAG-3*) [104], and Porcellato et al. (*CTLA-4*) [105]. The *RPS19* gene was used as a non-regulated reference (housekeeping) for normalization of the target gene expression. The relative mRNA expression was calculated using the comparative Ct method [106] as 2^−ΔCt^, (ΔCt  =  Ct_target_ − Ct_reference_). The sequences of all primers used for qPCR are listed in Table 2.

### 4.8. Statistical Analysis

Statistical analysis was performed using GraphPad PrismTM 5.0 (GraphPad Software Inc., San Diego, CA, USA). Normality was assessed using histogram plots and a Shapiro–Wilk test. Since all datasets followed a normal distribution, comparisons between multiple groups were performed using one-way analysis of variance (ANOVA) with Tukey’s multiple comparison test or two-way RM ANOVA with Bonferroni-corrected post hoc comparisons, as indicated in figure legends. Comparisons between two groups were analyzed using a two-tailed Student’s *t*-test (as indicated in the figure legends). A *p* value < 0.05 was considered statistically significant. Symbols indicate a significant difference between the indicated groups, as follows: * *p* < 0.05; ** *p* < 0.01; *** *p* < 0.001.

## Figures and Tables

**Table 1:** Morphological and biochemical parameters of shelter (short- and long-term stay) and client-owned dogs.

Parameters	Units	Values			
		Short-Term	Long-Term	Client-Owned	Reference Ranges
RBC	T/L	7.7 ± 0.64	7.21 ± 0.59	7.3 ± 0.38	5.2–7.9
HGB	g/dL	17.87 ± 1.72	16.9 ± 1.37	16.14 ± 1.34	12.4–19.2
HCT	%	48.93 ± 5.13	46.12 ± 3.44	43.85 ± 3.4	35.0–52
MCV	fL	63.03 ± 2.71	64.12 ± 2.42	62.78 ± 2.3	60.0–71
MCHC	g/dL	36.47 ± 1.75	36.01 ± 2.32	36.87 ± 1.6	34.4–38.1
PLT	G/L	303.33 ± 76.17	234.4 ± 73.71	262.85 ± 54.9	108–562
WBC	G/L	12.72 ± 2.56	11.1 ± 2.03	11.32 ± 2.61	6.00–17
NEU	G/L	7.13 ± 2.32	6.18 ± 1.43	6.48 ± 1.91	2.90–13.6
LYM	G/L	3.6 ± 1.46	3.01± 0.93	2.93 ± 0.97	1.10–5.3
MON	G/L	0.9 ± 0.26	0.8 ± 0.21	0.92 ± 0.25	0.40–1.6
EOS	G/L	0.72 ± 0.46	1.08 ± 0.6	0.87 ± 0.6	0.10–3.1
BAS	%	0.32 ± 0.22	0.22 ± 0.11	0.32 ± 0.17	0–1
ALT	U/L	32.04 ± 11.11	41.25 ± 10.22	36.75 ± 11.03	<60
AST	U/L	42.02 ± 20.51	38.87 ± 8.64	39.58 ± 13.53	<45
ALP	U/L	44.67 ± 24.29	62.54 ± 24.98	41.58 ± 18.21	<155
Urea	mg/dL	33.1 ± 8.33	33.17 ± 7.07	29.23 ± 5.93	20–50
Creatinine	mg/dL	0.97 ± 0.17	0.94 ± 0.16	0.87 ± 0.19	0.5–1.7
Total Protein	g/dL	6.81 ± 0.47	6.34 ± 0.32	6.68 ± 0.38	5.5–7.5
Albumin	g/dL	3.8 ± 0.3	3.86 ± 0.13	3.95 ± 0.15	3.3–5.6
Globulin	g/dL	2.82 ± 0.56	2.62 ± 0.49	2.72 ± 0.34	2.1–4.5
Lipase	U/L	82 ± 30	73 ± 34	63 ± 41	<120
Cortisol	μg/dL	1.4 ± 0.56	1.51 ± 0.34	1.3 ± 0.45	1–6
CRP	mg/L	<10	<10	<10	<20

**Table 2:** Sequence of primers used in real-time PCR.

Gene	Starters Sequence (F—Forward, R—Reverse)
*CD25*	F: 5′-ACTCCAGATTTCCACAAACACACA-3′ R: 5′-GCTCTTCTTGGCTTCTTACCACT-3′
*CD69*	F: 5′-AGGGTGCTACTCTTGCGTT-3′ R: 5′-CAGTAAGGTTGAGCCAGTTGC-3′
*IL-2*	F: 5′-ATCGCACTGACGCTTGTACTT-3′ R: 5′-GTGTAAATTCTGTGGCCTTCTTGG-3′
*IFN-γ*	F: 5′-TCAAGGAAGACATGCTTGGCAAGTT-3′ R: 5′-GACCTGCAGATCGTTCACAGGAAT-3′
*TNF-α*	F: 5′-CCAAGTGACAAGCCAGTAGC-3′ R: 5′-TCTTGATGGCAGAGAGTAGG-3′
*GZMA*	F: 5′-TGGTTCCTGGAGATTTCTGTG-3′ R: 5′-GTTTTTCCGACTCCTTCTTGG-3′
*GZMB*	F: 5′-CAAGATACGCAGGTACCCAGA-3′ R: 5′-TCCCTGAAAGGAAGACTTGGT-3′
*PRF1*	F: 5′-CAGGAGCAGAGAACCTACACG-3′ R: 5′-AGCACTTGGCAATGTAGGAGA-3′
*LAG-3*	F: 5′-AGTCATCACAGTGACTCCCA-3′ R: 5′-GAACCTCGCCAAGACTGCTT-3′
*CTLA-4*	F: 5′-GCCCTGCACTGCTCTGTTTT-3′ R: 5′-TCACACACGAAGCTAGCAACA-3′
*RPS19*	F: 5′-GTTCTCATCGTAGGGAGCAAG-3′ R: 5′-CCTTCCTCAAAAAGTCTGGG-3′

## Data Availability

The original contributions presented in this study are included in the article. Further inquiries can be directed to the corresponding author.
